# Reply to Kalfert, D. Comment on “Zwierz et al. The Long-Term Effects of 12-Week Intranasal Steroid Therapy on Adenoid Size, Its Mucus Coverage and Otitis Media with Effusion: A Cohort Study in Preschool Children. *J. Clin. Med.* 2022, *11*, 507”

**DOI:** 10.3390/jcm11092270

**Published:** 2022-04-19

**Authors:** Aleksander Zwierz

**Affiliations:** Department of Otolaryngology, Phoniatrics and Audiology, Faculty of Health Sciences, Ludwik Rydygier Collegium Medicum, Nicolaus Copernicus University, 85-168 Bydgoszcz, Poland; aleksanderzwierz@gmail.com

We wish to thank the author for raising the issues of how we performed the adenoid size classification and why we did not classify the condition of the nasopharyngeal orifice of the Eustachian tube [1].

The aim of our work, “The Long-Term Effects of 12-Week Intranasal Steroid Therapy on Adenoid Size, Its Mucus Coverage and Otitis Media with Effusion: A Cohort Study in Preschool Children”, was to assess the change in adenoid size following intranasal steroid treatment [2]. Therefore, we assessed the long-term effects of intranasal steroid therapy on adenoid size, as measured using the percentage scale of nasopharyngeal obstruction (adenoid to choana (A/C) ratio). The size of the tonsil was analysed in relation to the height of the nasopharynx in a similar way to the assessment proposed by Wormald and Prescott [3]. To achieve a better statistical assessment, the patients were divided into groups, which we determined using the three-step Bolesławska scale, particularly the part regarding the size of the tonsil in relation to the nasopharynx [4]. This approach is described in detail in the Material and Methods section of the manuscript. 

It is well-known that there are several similar classifications available for assessing the size of the tonsil in relation to the nasopharynx. For example, Cassano et al. proposed a four-step pictorial scheme for classifying nasopharyngeal obstruction by the tonsil (occupying 0–25%, 26–50%, 51–75% or 76–100% of the nasopharynx) [5]. Moreover, Zalzal et al. proposed a five-step scale for assessing adenoid size, wherein grade 0 indicates 0% obstruction of the choanae, grade 1 indicates less than 40% obstruction, grade 2 indicates 41–70% obstruction, grade 3 indicates 71–90% obstruction and grade 4 indicates complete obstruction (91–100%) of the choanae with lymphoid tissue touching the soft palate when at rest [6]. It should be noted that we primarily presented an accurate percentage analysis of the change in the adenoid and, further, that we used the secondary part of the Bolesławska scale as a means of simplification for the purposes of statistical analysis. 

Our goal was to assess the change in the size of the tonsil as a result of treatment with intranasal steroids, which could indirectly cause anatomical changes in the structure of the nasopharynx. However, we did not analyse in detail the change in the relationship between the tonsil and the torus tubarius or salpingopharyngeal and salpingopalatine folds. Thus, we did not analyse the anatomical part of the Bolesławska scale. For this purpose, we preferred to use the newer scale proposed by Liu et al. [7]. Yet, we have not confirmed the long-term effects of intranasal steroid treatment on otitis media with effusion OME, which may suggest that the anatomical relations between adenoid and nasopharynx structures have not been altered. This issue requires further analysis and investigation in accordance with Liu classification because even a small change in adenoid size may be influenced by its relationship to the Eustachian tube. As described in studies conducted by Skoloudik and Hazem, anatomical relations of the adenoid with the torus tubarius may influence the results of OME treatment involving adenoidectomy, although we would like to point out that there are many other factors that can also affect OME, for example, adenoid mucus coverage and seasonality [8,9,10]. Nevertheless, this issue was not a focus of our work, as we analysed the long-term effects of intranasal steroid treatment on OME.

Finally, we would like to add that we chose to use the Bolesławska scale in order to promote the researcher’s work. It should be noted that the work and classification were published in Czech in the same year (2006) as the similar but much better-known Parikh classification [11]. In parts, both of these classifications concern the anatomical relations of the tonsil with the structure of the nasopharynx.

## References

[1] Kalfert D. (2022). Comment on Zwierz et al. The Long-Term Effects of 12-Week Intranasal Steroid Therapy on Adenoid Size, Its Mucus Coverage and Otitis Media with Effusion: A Cohort Study in Preschool Children. *J. Clin. Med.* 2022, *11*, 507. J. Clin. Med..

[2] Zwierz A., Masna K., Domagalski K., Burduk P. (2022). The long-term effects of 12-week intranasal steroid therapy on adenoid size, its mucus coverage and otitis media with effusion: A cohort study in preschool children. J. Clin. Med..

[3] Wormald P.J., Prescott C.A. (1992). Adenoids: Comparison of radiological assessment methods with clinical and endoscopic findings. J. Laryngol. Otol..

[4] Boleslavská J., Koprivová H., Komínek P. (2006). Is it important to evaluate the size of adenoid vegetations?. Otorinolaryng Foniat Prague.

[5] Cassano P., Gelardi M., Cassano M., Fiorella M.L., Fiorella R. (2003). Adenoid tissue rhinopharyngeal obstruction grading based on fiberendoscopic findings: A novel approach to therapeutic management. Int. J. Pediatric Otorhinolaryngol..

[6] Zalzal H.G., Carr M., Nanda N., Coutras S. (2018). Drug induced sleep endoscopy identification of adenoid regrowth in pediatric obstructive sleep apnea. Int. J. Otolaryngol..

[7] Liu H., Feng X., Sun Y., Fan Y., Zhang J. (2021). Modified adenoid grading system for evaluating adenoid size in children: A prospective validation study. Eur. Arch. Oto-Rhino-Laryngol..

[8] Skoloudik L., Kalfert D., Valenta T., Chrobok V. (2018). Relation between adenoid size and otitis media with effusion. Eur. Ann. Otorhinolaryngol. Head Neck Dis..

[9] Abdel Tawab H.M., Tabook S.M.S. (2021). Correlation between Adenoid Hypertrophy, Tympanometry Findings, and Viscosity of Middle Ear Fluid in Chronic Otitis Media with Effusion, Southern Oman. Ear Nose Throat J..

[10] Masna K., Zwierz A., Domagalski K., Burduk P. (2021). The impact of the thermal seasons on adenoid size, its mucus coverage and otitis media with effusion: A cohort study. J. Clin. Med..

[11] Parikh S.R., Coronel M., Lee J.J., Brown S.M. (2006). Validation of a new grading system for endoscopic examination of adenoid hypertrophy. Otolaryngol. Head Neck Surg..

